# Spatiotemporal analysis of tropical disease research combining Europe PMC and affiliation mapping web services

**DOI:** 10.1186/s41182-017-0073-6

**Published:** 2017-10-26

**Authors:** Magnus Palmblad, Vetle I. Torvik

**Affiliations:** 1 Center for Proteomics and Metabolomics, Leiden University Medical Center, Leiden, the Netherlands; 2 School of Information Sciences, University of Illinois at Urbana-Champaign, Champaign, IL USA

## Abstract

**Background:**

Tropical medicine appeared as a distinct sub-discipline in the late nineteenth century, during a period of rapid European colonial expansion in Africa and Asia. After a dramatic drop after World War II, research on tropical diseases have received more attention and research funding in the twenty-first century.

**Methods:**

We used Apache Taverna to integrate Europe PMC and MapAffil web services, containing the spatiotemporal analysis workflow from a list of PubMed queries to a list of publication years and author affiliations geoparsed to latitudes and longitudes. The results could then be visualized in the Quantum Geographic Information System (QGIS).

**Results:**

Our workflows automatically matched 253,277 affiliations to geographical coordinates for the first authors of 379,728 papers on tropical diseases in a single execution. The bibliometric analyses show how research output in tropical diseases follow major historical shifts in the twentieth century and renewed interest in and funding for tropical disease research in the twenty-first century. They show the effects of disease outbreaks, WHO eradication programs, vaccine developments, wars, refugee migrations, and peace treaties.

**Conclusions:**

Literature search and geoparsing web services can be combined in scientific workflows performing a complete spatiotemporal bibliometric analyses of research in tropical medicine. The workflows and datasets are freely available and can be used to reproduce or refine the analyses and test specific hypotheses or look into particular diseases or geographic regions. This work exceeds all previously published bibliometric analyses on tropical diseases in both scale and spatiotemporal range.

## Background

Tropical medicine first appeared as a distinct sub-discipline and professional specialization toward the end of the nineteenth century, and the heyday of tropical medicine coincided with European colonialism in Africa and Asia around this time. After the decades following World War II, recent years have seen an increasing attention and significant funding to combat tropical diseases in an increasingly globalized world. In this paper, we attempt to visualize these and other aspects of the history of tropical medicine by spatiotemporal bibliometric analyses.

This is not the first bibliometric venture into the history of research on tropical diseases. In 2006, Falagas et al. published two studies [1, 2] on parasitology and tropical medicine research respectively over the 9-year period 1995–2003 identifying Oceania countries as the most productive when adjusting for both gross national income per capita and population. The authors also noted the number of publications on parasitology from Latin America, the Caribbean, and Asia doubled between 1995 and 2003, but that the production from African countries remained low despite many of the diseases being endemic here. More recently, Ramos et al. published a bibliometric analysis of Chagas disease research 1940–2009 [3] and leishmaniasis research 1945–2010 [4], identifying Brazil as the most productive country in the first decade of the twenty-first century when looking at the first-author affiliations. Similarly and more recently, Zyoud et al. published a spatiotemporal bibliometric analysis of publications on dengue 1872–2015 [5], noting both the most productive countries in the field and a considerable increase in dengue-related publication in the last decade. For Sub-Saharan Africa [6–8] and Latin America [9], biomedical research, including neglected infectious diseases and the relation between disease burden and clinical trials, has been assessed by bibliometric methods. The field has seen rapid development in recent years, and an updated analysis of research output on tropical diseases is therefore motivated. What are the global and historical trends in tropical medicine research, and how do recent outbreaks, attention, and funding compare in these contexts? What else can be learned from broad, spatiotemopral bibliometric analyses?

Here, we also show how to use scientific workflows and freely available web services for spatiotemporal bibliometric analyses. Scientific workflows integrate specialized software, databases, or services into an overall data flow. They are particularly well suited for multi-step analyses using different types of software tools. The workflows are reusable for similar purposes and make analyses reproducible. Using web services and online databases, the workflows always access the latest information. Technical details on how the literature and geoparsing web services are accessed and the returned data parses are abstracted and tucked away in workflow components, allowing less experienced users to focus on the overall workflow logic and scientific hypothesis. To our knowledge, this is the first time literature and geoparsing web services have been integrated this way. The bibliometric analyses were done in Taverna workflows available on myExperiment [10].

## Methods

**Fig. 1:**
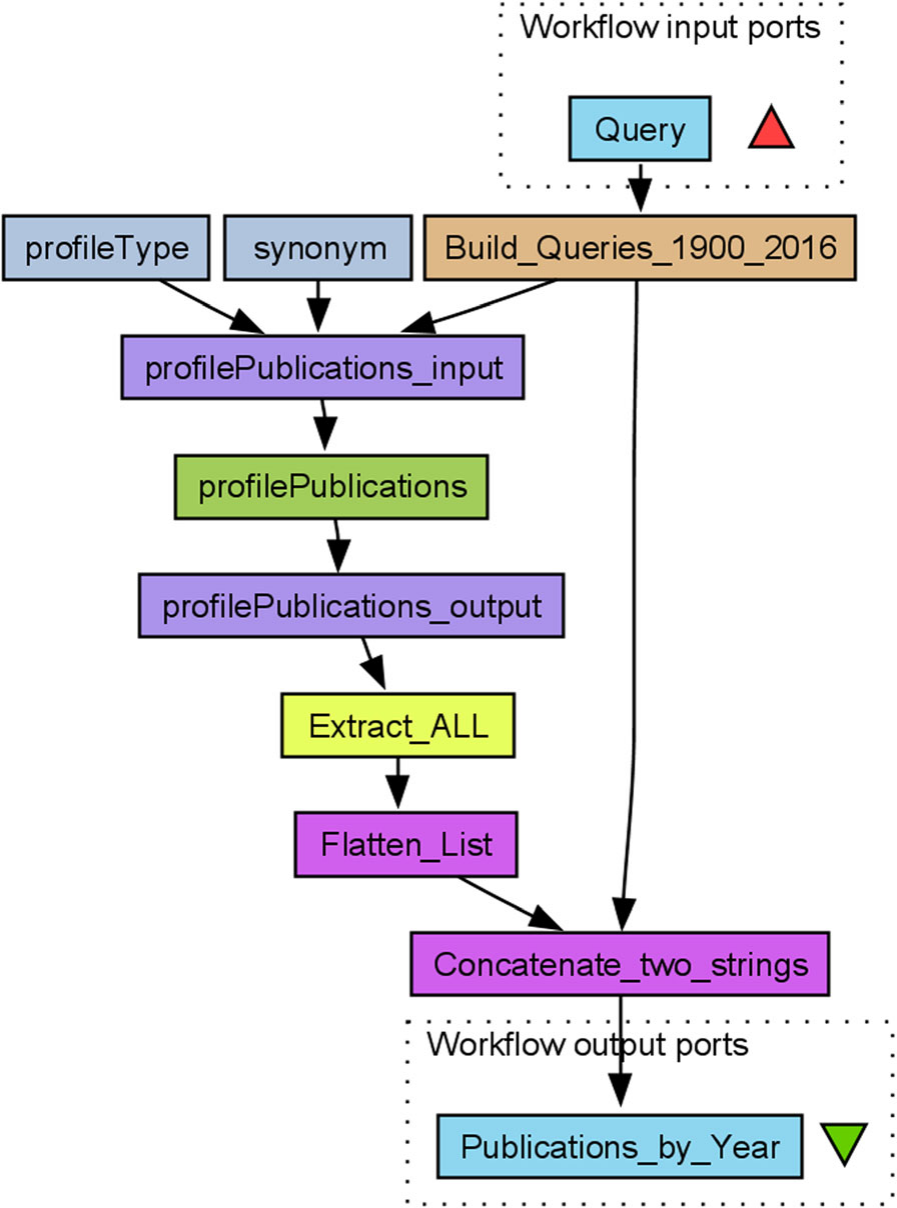
The *profilePublications_over_time* workflow. This *profilePublications_over_time* Taverna workflow used to collect publication year data on publications of all types and on the topic defined by the input *Query*

To count the number of publications on specific topics, such as a disease, we used the Europe PubMed Central (PMC) *profilePublications* Simple Object Access Protocol (SOAP) web service [ref. Europe PMC or manual]. This service returns summaries by category, i.e., database source (Agricola, CiteXplore, PubMed/MEDLINE NLM, PubMed Central, Biological Patents, etc.) and publication type (full text, open access, reviews, books, and documents). A Taverna [11] workflow *profilePublications_over_time* integrating this service is shown in Fig. 1 and also shared on the myExperiment [10] website (http://myexperiment.org/workflows/4980.html). This workflow takes as input one or more Europe PMC search queries. From each of these, the *Build_Queries_1900_2016* component generates 117 individual search queries to retrieve the publication summaries by year. The *Extract_ALL* conditional XPath extracts the total number of publications in all categories. The workflow outputs a list with the number of publications per year, similar to the “Results by year” chart in the PubMed web interface. We ran this workflow in Taverna Workbench Core 2.5.0 in November 2016, with a list of the 10 most researched tropical diseases as defined by WHO (malaria, cholera, leprosy, schistosomiasis, trypanosomiasis, dengue, leishmaniasis, Chagas disease, Ebola, and taeniasis/cysticercosis).

For a spatiotemporal analysis of the scientific literature, in particular using PubMed and other open resources, it is often necessary to parse the author affiliation information. We performed this geoparsing using MapAffil [12], a tool specifically developed to parse the author affiliation strings in PubMed. MapAffil correctly identifies cities (or similar localities) and assigns the city-center geocodes to about 98% of affiliations in PubMed. The remaining 2% largely lack place information (e.g., only the name of a multi-location institution is given), while errors and unresolved ambiguities are rare.

**Fig. 2:**
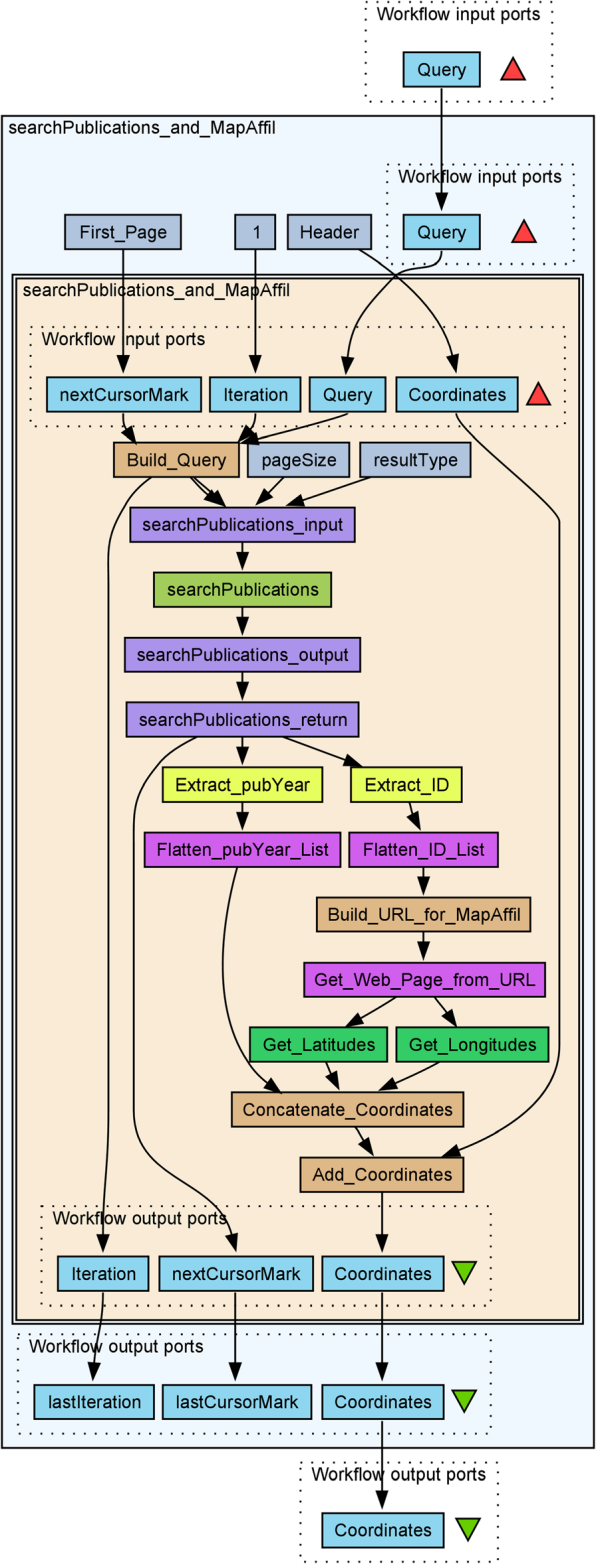
The *searchPublications_and_MapAffil* workflow*.* The Taverna workflow *searchPublications_and_MapAffil* combining the Europe PMC *searchPublications* web service with the MapAffil geoparser to map publications in Europe PMC on a given topic. The innermost workflow is embedded for the looping construct in Taverna in order to retrieve all *searchPublication* results pages, *pageSize* = 100 records as a time for MapAffil. The *cursorMarks* were introduced in version 4.5.3 of the web services to robustly handle multiple results pages. Older workflows with offsets [13] no longer work with these services. The workflow is available on myExperiment (http://myexperiment.org/workflows/4981.html)

A Taverna workflow *searchPublications_and_MapAffil* integrating the Europe PMC *searchPublications* SOAP web service as previously described [13, 14] with MapAffil using its REST-like API is shown in Fig. 2. The workflow takes as input one or more queries, searches Europe PMC using *searchPublications*, retrieve the records and extract PubMed IDs and publication years with two XPaths. The IDs are passed to MapAffil by *Build_URL_for_MapAffil* and the built-in *Get_Web_Page_from_URL service*. MapAffil returns geoparsed affiliations with official city, county and country names, FIPS codes, latitudes and longitudes in WGS 84, and author orders in JSON. Two JsonPaths, *Get_Latitudes* and *Get_Longitudes*, extract coordinates for the first authors. The coordinates are then combined into one list of latitudes, longitudes, and publication years. The workflow outputs this list and *hitCount*, the number of records returned from Europe PMC, for each input query. The workflow was run in November 2016, using the same input as the first workflow.

Geographical information can be visualized using different software tools, including from within Taverna using the rworldmap [13, 15] or RQGIS [16] R packages. Here, we used the standalone Quantum Geographic Information System (QGIS) [17] desktop software version 2.18.0 and directly imported the coordinates from the *searchPublications_and_MapAffil* workflow in Fig. 2 as a delimited text layer in QGIS and overlaid these on a world map. For co-authorship analysis, we used VOSviewer [18] version 1.6.5 and projected the collaborative network, using latitude and longitudes from MapAffil, but for all co-author affiliations, onto the same world map. Collaborative clusters were extracted using resolution = 0.3 and minimum size = 100. These parameters determine the sensitivity for separating clusters, and how many nodes are required to form a unique cluster.

## Results and discussion

**Fig. 3:**
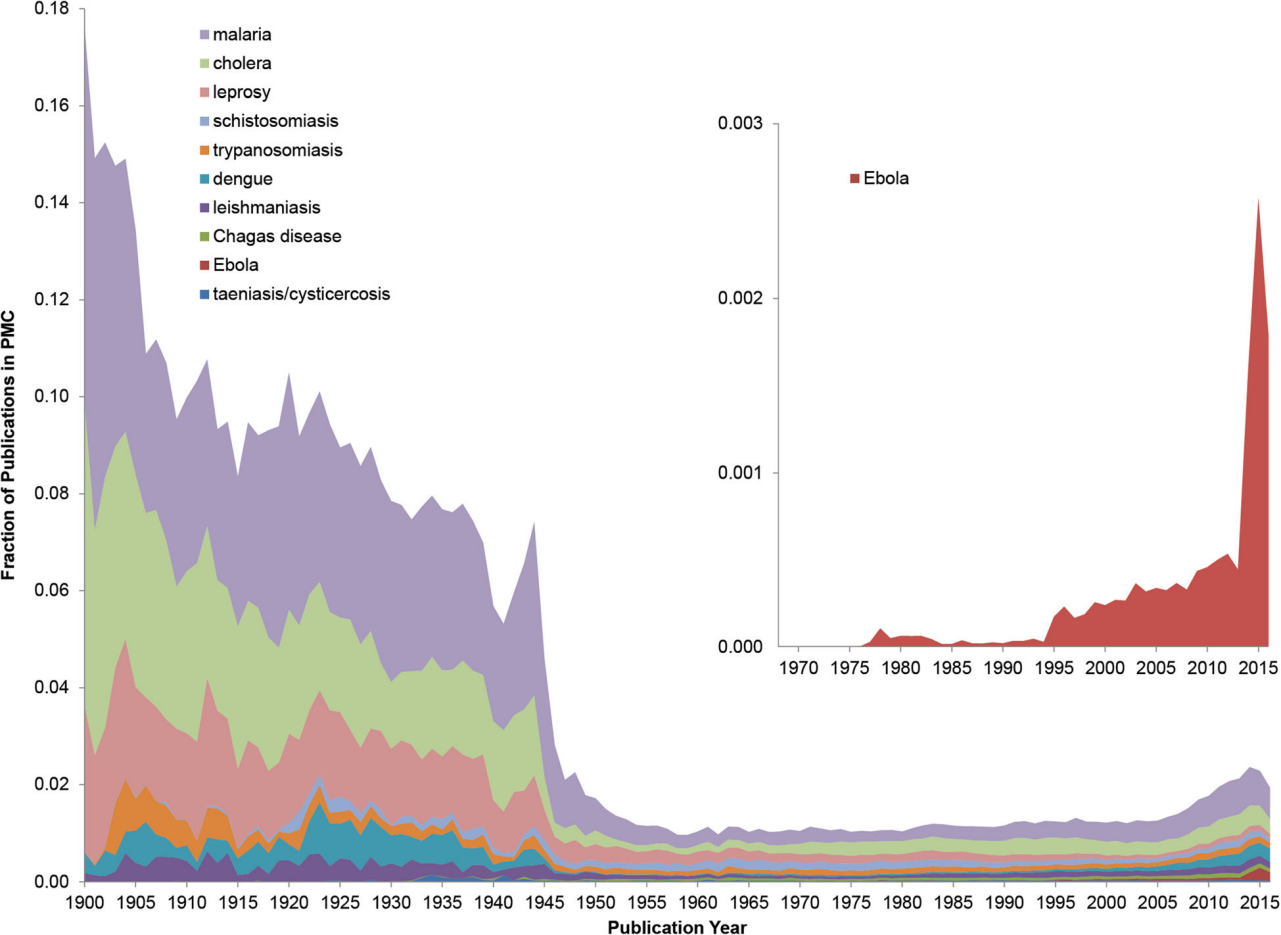
Publication on tropical diseases 1900–2016. Publications on the 10 most published on tropical diseases between 1900 and 2016. The magnified region in the inset shows the relative number of publications on Ebola since the first outbreak in 1976

The results from the *profilePublications_over_time* workflow are summarized in Fig. 3. The figure shows the fraction of publications in PubMed devoted to one or more of these tropical diseases over the course of the twentieth and the early twenty-first century. This fraction was highest in the early 1900s (for malaria in 1900–1901, trypanosomiasis 1903–1904, and leishmaniasis 1912–1914). Minor dips can be observed at the beginning of World War II in 1939–1940 and to a lesser degree after the outbreak of World War I in 1914–1915. The absolute number of papers in PubMed increase dramatically after 1945, whereas the absolute number of publications per year on tropical diseases grew more slowly (malaria), remained largely constant (cholera), or even decreased (dengue fever). To some degree, this may have been a direct consequence of the rapid decolonization of Africa, South, and Southeast Asia after 1945, reducing the relative research output on tropical diseases from the UK, France, Belgium, and the Netherlands. In this century, with increased emphasis on previously neglected tropical diseases and research funding, for example, though the Bill & Melinda Gates Foundation since 2000, the fraction of publications on tropical diseases has slowly increased since around 2005–2007. For Ebola, which is a special case, we observe (Fig. 3, inset) an initial local maximum in 1978, 2 years after the first outbreak in Zaire September-October 1976, with interest waning around 1983–1984. After a period of few publications per year, we note a larger increase in 1995 after the outbreak the same year in DRC. The research output then increased steadily, in particular after the outbreaks in Congo and Uganda 2000–2001, and spiked in 2015–2016 after the major Uganda outbreak in 2014. Each time, the outbreak was followed by a distinct increase in research output. Although seven Ebola articles were published in 1977, within a year of the first outbreak, later outbreaks were followed by a more rapid increase in the number of research publications.

Fewer publications on a particular research topic or disease do not imply neglect. Though not exclusively a tropical disease, smallpox was successfully eradicated in 1980. This is clearly seen in the research output, where a period of higher research output with 309 ± 42 publications per year during the WHO Smallpox Eradication Programme 1966–1980 followed by a period of lower output with 129 ± 19 publications per year between 1981 and 1995. With increasing concerns of bioterrorism in the early 2000s, the number of publications increased dramatically, reaching maximum of 756 publications in 2003. Similar trends can be observed for polio, with an increased research output from 1952, the year the first successful vaccine was developed, reaching a local maximum of 326 publications in 1957, and then falling as the incidence declined rapidly following mass vaccination in developed countries, until reaching a steady level of ~ 150 publications/year from the mid-1960s until the mid-1980s.

**Fig. 4:**
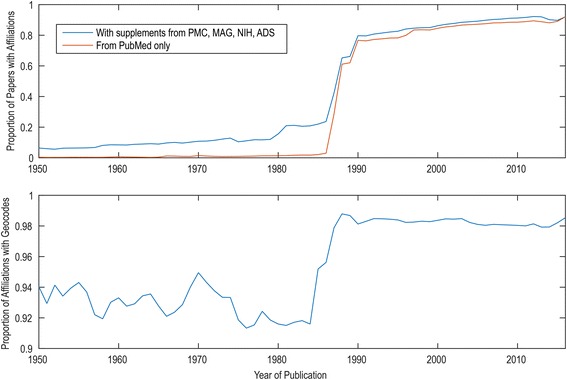
MapAffil coverage. Coverage of MapAffil over time, showing the fraction of papers for which affiliation information is available (*top*) and the fraction of these with MapAffil geocodes (*bottom*). This poses a challenge, especially for narrow historical analyses going back further than 1990, and explains why much published bibliometrics work begin between 1990 and 1995

Geoparsing PubMed affiliations reveals *where* research was conducted, in addition to *when*. Table 1 contains the number of results, first authors, and mapped first-author affiliations for the 10 tropical diseases. In total, the workflow retrieved 379,728 records on these 10 tropical diseases. Only 259,138 or 68.2% of the retrieved Europe PMC records have a first-author affiliation explicitly designated as such. This information is lacking in many older publications (and we are here looking at publications as far back as the early 1800s, at least for leprosy and malaria). However, from these, 253,277 or 97.7% could be successfully parsed to geographical coordinates by MapAffil. We did not observe a major difference in the MapAffil success rate between diseases (range from 95.9 to 98.3%) or as a function of publication year. Temporal artefacts may be due to the availability of affiliations in PubMed, with indexing starting in 1987 and all authors’ affiliations being available only from 2014. Publications older still are available for specific journals, such as *Medico-Chirurgical Transactions* (1809–1907)/*Proceedings of the Royal Society of Medicine* (1908–1977) provided by the Royal Society of Medicine Press, and journals such as *British Medical Journal* (1857–1980), *Journal of Anatomy and Physiology* (1867–1916), *The Journal of Physiology* (1878-), and *Annals of Surgery* (1885-). Even though single-author publications were common in the past, there may still be some geographic bias from looking only at the first authors of older papers. For example, looking at all authors may reveal additional insights into the structure of research collaboration and accurately cover field work and local collaborators in endemic areas. The last author/principal investigator affiliation may correlate with the institution awarded the grants to conduct the research. The overall coverage of affiliations in MapAffil over time is shown in Fig. 4. The US National Library of Medicine started consistently recording first-author affiliations in 1988. Some of the records lacking affiliations were supplemented with data harvested from sources external to PubMed, including PMC, Microsoft Academic Graph (MAG), US National Institutes of Health (NIH) grants, and the Astrophysics Data System (ADS). For MAG and ADS, a crosswalk between citations was created using the Patci citation matcher [19], while NIH grant links were based on grant numbers listed in the XML distribution of PubMed. As the trend in the figure shows, this supplementation has the greatest effect for papers published before 1985, where the coverage goes from ~ 0% to 10–20%. Since 1990, supplementation covers another 2% of papers, yielding ~ 80% coverage in 1990, and ~ 90% more recently. It should be noted that the figure does not reflect that supplemental affiliations are added to authors beyond the first author, which may pick up additional geocodes for multisite collaborations.

**Table 1:** The 10 most researched tropical diseases

Tropical disease	hitCount	1st authors	Mapped affiliations
Malaria	130,964	95,155	93,115
Cholera	62,697	40,586	39,742
Leprosy	41,979	20,254	19,741
Schistosomiasis	31,459	18,844	18,421
Dengue	30,554	24,446	23,800
Leishmaniasis	30,114	23,164	22,765
Chagas disease	17,185	12,966	12,714
Trypanosomiasis	15,381	9765	9532
Ebola	11,132	8997	8626
Taeniasis/cysticercosis	8263	4961	4821

The 10 tropical diseases (WHO definition) most published on, as indexed by Europe PMC, with the number of hits returned by *searchPublications_and_MapAffil*, the number of the first authors and the number of the first-author affiliations geoparsed by MapAffil

**Fig. 5:**
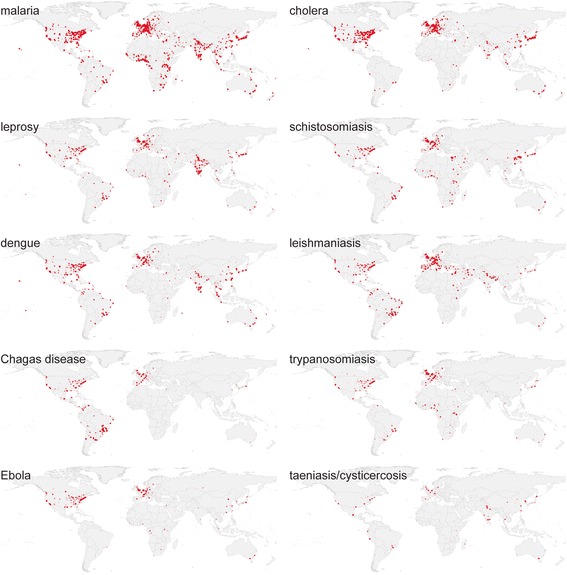
Tropical diseases research 1813–2016. Map of absolute research output on tropical diseases 1813–2016 measured by 253,277 publications in Europe PMC and geoparsed by MapAffil (all on the same QGIS linear heatmap scale with radius 1.25 and maximum value 25). Made with Natural Earth. Free vector and raster map data at naturalearthdata.com

The spatiotemporal analysis by the *searchPublications_and_MapAffil* workflow shows the expected correlation between disease prevalence and research output. For example, Fig. 5 shows the geographical distribution of research output for the 10 tropical diseases based on all publications in PubMed 1813–2016 for which the first-author affiliation was available and could be geoparsed. In absolute terms, Western Europe and North America dominate research output on most tropical diseases except leprosy and Chagas disease. The geographic differences become more pronounced when looking at low- and medium-income countries, many of which are affected by one or more of these tropical diseases. South-East Asia (including India) is clearly overrepresented in research output on leprosy. This is consistent with disease prevalence and a previous report by Schoonbaert and Demedts [20]. In 2014, 72% of all reported new cases were detected in this region [21]. Research output on Chagas disease, also known as American trypanosomiasis, is concentrated in South America (Brazil in particular). We also observe a high proportion of research output on malaria in all tropical regions. Dengue research is found more broadly over South and Southeast Asia, and Leishmaniasis research is concentrated in the Middle East (Israel, Egypt) and Brazil. A significant share of the funding Egypt and Israel received after the 1978 Camp David accord went to malaria and leishmaniasis research [22]. Ramos et al. [4] have also found that Israel produced the largest number of publications on leishmaniasis per capita. On the other hand, Ebola research output is concentrated to a few hotspots in Africa, such as Franceville, Brazzaville, Kampala, and Nairobi. When filtered by publication year, it is clear that the fraction of research publication coming from outside Europe, North America, or Japan increases dramatically after World War II. When analyzing these maps in detail, it should be remembered that fieldwork is, by definition, geographically separated from the research institutes often in the author affiliations.

**Fig. 6:**
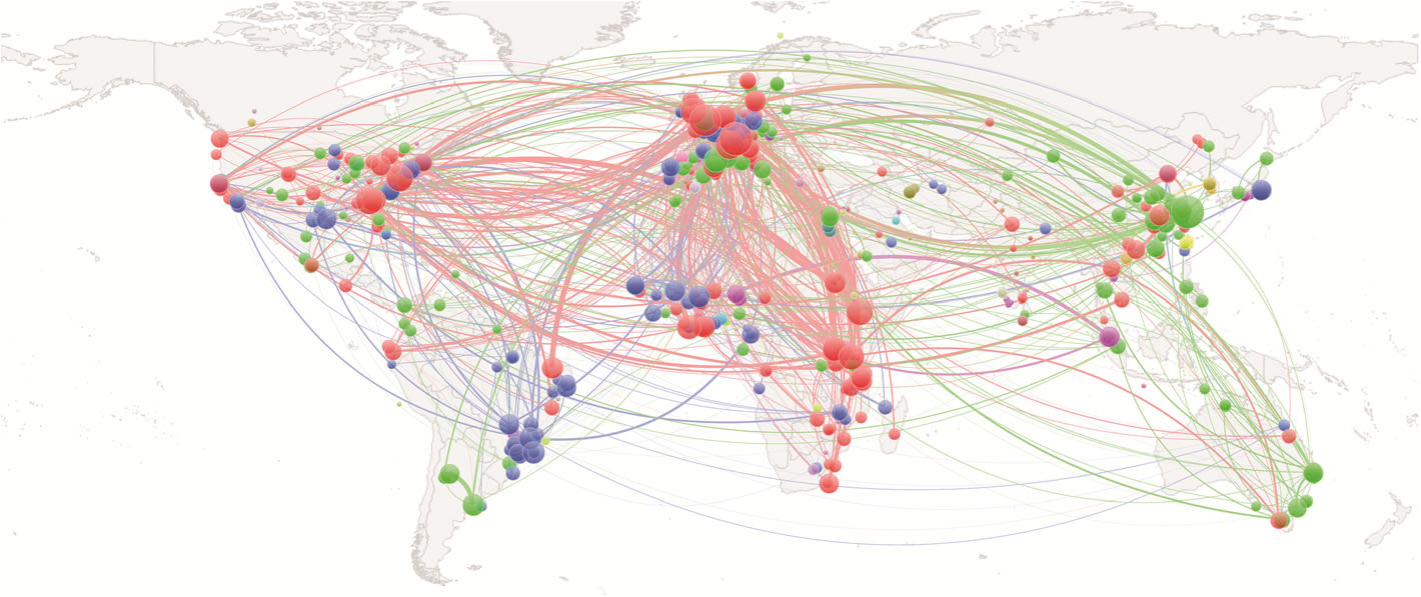
Map of current schistosomiasis research collaborations. Geographical patterns of collaboration based on co-authored publications on schistosomiasis in 2016. Made with Natural Earth (free vector and raster map data at naturalearthdata.com). The colors indicate clusters as assigned by the VOSviewer. Nodes within a cluster have stronger coupling with other nodes in the same cluster than with nodes in other clusters

Figure 6 shows the geographical patterns of schistosomiasis research, based on co-authorships in Europe PMC with the first publication date in 2016. Although the field is highly internationalized, the collaborations separate in clusters, where cluster 1 (red, 199 locations) is dominated by the UK and anglophone countries in East Africa. Cluster 2 (green, 141 locations) dominated by Asia, and cluster 3 (blue, 125 locations) shared between France (Paris, Lille, Caen, Bordeaux) and francophone countries in West Africa, Portugal, and Brazil. This is consistent with the social network analysis of international academic ties by Safonova and Sokolov [23], identifying “academic neocolonialism” being of primary importance for institutional links and the collaborative clusters they form, and explaining observed patterns of international student flows. Lacunae, “missing” research output from major countries with high disease prevalence, may be due to conditions disadvantageous for conducting research, such as civil war or unrest, or lack of infrastructure. This is difficult to quantify, as also disease prevalence may be underreported from such regions. Affiliations for authors other than the first are only available in for very recent publications, and historical studies on international collaborations are for this reason difficult in any field. The recent relationship between developed and developing countries has recently been investigated by González-Alcaide and co-workers [24], showing that countries of low and medium income have a higher degree of participation in areas of tropical medicine (co-authorship of 41% of research publications) and parasitology (24%) than infectious disease (19%) or pediatrics (8%) between 2011 and 2015.

We here used simple search queries and disabled the synonym lookup options in the Europe PMC web services. This will result in the inclusion of a few unrelated publications; for example, one paper from 1958 [25], 18 years before the first report, the Ebola *hemorrhagic fever*, on the geographic distribution of endemic goiter, including the areas watered by the Ebola *river*. Topic disambiguation is possible using Medical Subject Headings (MeSH). For example, Ramos et al. in their work [4] looked for the MeSH terms “Leishmania” or “leishmaniasis.” Using MeSH may also bridge publications that exclusively refer to a disease by an alternate name, such as leprosy as Hansen’s disease or schistosomiasis as bilharziasis or Katayama fever, though care should be taken that all synonyms are specific and that searches for all diseases are expanded to a similar “depth.” Text-mining methods can also be used to disambiguate topics de novo but will only be usefully accurate for full-text articles. Regardless of query specification, some relevant articles will always be missed, and some less relevant included, in large datasets.

## Conclusions

This paper illustrates how literature search and geoparsing web services can be combined in scientific workflows for reproducible, shareable, and reusable spatiotemporal bibliometric analyses. We have demonstrated this using research on 10 tropical diseases, as these exhibit characteristic and interpretable spatiotemporal patterns. Other resources that could, in principle, be combined in similar workflows include, for example, genomic, molecular, and epidemiological data, though geographical mapping of disease is a challenging but rapidly progressing field in itself [26–28]. The European Nucleotide Archive, ENA, and UniProt are extensively linked with publication Europe PMC. These database links can also be traversed using the *searchPublications* and *getDatabaseLinks* web services from Europe PMC and RESTful web services from UniProt.

Research output on tropical diseases has some correlation with disease burden, in particular when comparing countries of similar resources and research output. Shared colonial history and language are also important factors. The Ebola example suggests the research community now reacts faster and more strongly than the past decades upon outbreaks of diseases in Sub-Saharan Africa.

All work was performed on open data using freely available tools, including Taverna Workbench, Europe PMC, MapAffil web services, and QGIS. The two workflows are available from myExperiment for anyone who wishes to repeat or modify our analyses, without the need to download any bibliographic databases. The workflows and results are also available on the Open Science Framework (osf.io/dtkep/).

## Abbreviations

ADS: Astrophysics Data System
ENA: European Nucleotide Archive
JSON: JavaScript Object Notation
MAG: Microsoft Academic Graph
MeSH: Medical Subject Headings
NIH: National Institutes of Health
NLM: National Library of Medicine
PMC: PubMed Central
QGIS: Quantum Geographic Information System (now QGIS)
REST: REpresentational State Transfer
SOAP: Simple Object Access Protocol (now SOAP)
VOS: Visualization Of Similarities
WHO: World Health Organization
XML: Extensible Markup Language
